# The use of a psychological testing instrument as an indicator of dissatisfaction with aesthetic dental treatment – a preliminary study

**DOI:** 10.1186/s40359-020-0391-z

**Published:** 2020-03-14

**Authors:** James Dudley, Lindsay Richards, Melati Mahmud

**Affiliations:** 1 Adelaide Dental Hospital, The University of Adelaide, Adelaide, South Australia Australia; 2 Faculty of Dentistry, Universiti Teknologi Mara, Sungai Buloh, Selangor Malaysia

**Keywords:** Patient dissatisfaction, Psychological testing, Aesthetic dental treatment

## Abstract

**Background:**

The use of psychological testing to indicate the potential for dissatisfaction with dental treatment has many potential patient and clinician benefits but has been rarely investigated. The study aimed to explore the use of the Symptom Checklist-90-Revised (SCL-90-R) psychological testing instrument in describing the relationship between pre-treatment psychological traits and aesthetic restorative treatment satisfaction.

**Methods:**

Thirty patients requiring aesthetic restorative dental treatment completed three questionnaires, namely 1) a pre-treatment expectation assessment, 2) an SCL-90-R analysis pre-treatment and 3) an outcome assessment post-treatment to assess patient’s expectations and satisfaction of the proposed dental treatment relating to function, aesthetics, comfort and tissue preservation. Logistic regression models were used to assess the impact of psychological variables on patient satisfaction after adjusting for baseline expectations (*P* < 0.05).

**Results:**

The satisfaction for the aesthetic component of treatment was significantly associated with psychoticism and positive symptom distress index. The satisfaction for the comfort component of treatment was significantly associated with obsessive compulsive symptoms, depression and anxiety. Following adjustment for baseline expectation, tissue preservation satisfaction was associated with somatization, obsessive compulsive, interpersonal sensitivity, depression and global severity index. No baseline psychological measures were significantly associated with chewing satisfaction.

**Conclusions:**

The SCL-90-R shows initial promise in assisting clinicians to identify and understanding patients who have a high risk of dissatisfaction with aesthetic dental treatment. The ability to indicate aesthetic restorative treatment dissatisfaction is of great benefit to clinicians in maximising success and mitigating risk.

## Background

Patient satisfaction is a critical factor in determining the outcome of dental treatment. While clinicians may consider comfort, function and aesthetics as key treatment goals, patient satisfaction with dental treatment has been linked to technical practice, convenience and interpersonal interactions [1].

Despite advances in technology and dental materials, there has been relatively little focus on the patient psychological dimension of dental treatment that includes dimensions such as depression, obsessive compulsive and somatization [2]. Previous associations have been found between patient psychological traits and myofascial pain, denture incompatibility and aesthetic restorative treatment with dissatisfaction after aesthetic restorative treatment specifically linked to the psychological dimension of neuroticism [2–4].

In an epidemiological study, 25% of 25–45 year old subjects were reported with a psychological disorder and 12.5% were in need of psychotherapeutic treatment [3]. According to the Australian Bureau of Statistics (2015), there were 4 million Australians (7.5% of the population) with psychological conditions [5]. There is a realistic possibility that a proportion of these people present to our dental practices.

Obvious signs of psychological disorders include symptoms such as major depression, societal withdrawal, mania and panic disorder. Some disorders are characterized by functional deterioration such as in schizophrenia, somatization disorder and anti-social personality [6]. In some cases, a dental clinician may not be aware that the patient has a psychological disorder as the symptoms at presentation may not always be straightforward and can be missed if not suspected and specifically explored.

The early recognition of psychological characteristics in the patient-dentist relationship is important to prevent patient disappointment in dental treatment regardless of the type of treatment [7]. A failure to meet expectations can create substantial costs and considerable non-productive time both for the clinician and the patient, as well as possible litigation that may follow. It would therefore be ideal to develop an inconspicuous method of assessment that could accurately identify patients for which satisfaction with dental treatment could be a problem.

In the medical field, clinical and self-report questionnaires have been utilised for the psychometric analysis of psychological inpatients [8]. NEO Five-Factor Inventory (NEO-FFI) has been reportedly used, although the investigated components are not as diverse as those contained in the Symptom Checklist-90-Revised (SCL-90-R). In dentistry, there is a dearth of evidence that has compared the various psychological testing instruments. The SCL-90-R is one questionnaire that has been used reliably to evaluate a broad range of psychological problems, although its use has not been reported in patients who had undergone aesthetic dental treatment [2–4, 9]. The aim of this study was to explore the use of the SCL-90-R psychological testing instrument in describing the relationship between pre-treatment psychological traits and aesthetic restorative treatment satisfaction.

## Methods

### Study location

This longitudinal cohort study was conducted in the undergraduate teaching clinics and specialist restorative unit of the Adelaide Dental Hospital (ADH) involving treatment provided by undergraduate students (under the supervision of qualified dentists), dentists and specialists. Ethics approval was obtained from the University of Adelaide Human Research Ethics Committee and the Central Adelaide Local Health Network / South Australian Dental Service (HREC/15/RAH/399).

### Participants

A convenience sample (*n* = 30) of patients aged 20–80 years requiring aesthetic restorative dental treatment including porcelain laminate veneers, composite resin restorations, intra-coronal bleaching of root filled teeth, single tooth implants and full coverage crowns was recruited from the ADH. The sample was representative of the number of patients who presented to a public dental clinic and were approved to receive aesthetic dental treatment. Patients were recruited as they presented to one of three clinics (undergraduate, postgraduate and prosthodontics) without randomisation.

Following treatment plan finalisation, each patient was provided with an information sheet and invited to take part in the study. Written informed consent was obtained when the patient agreed to participate in the study. Patients were excluded if they had an intellectual disability, severe medical conditions or were not fluent in English and could not understand or complete the questionnaires.

### Study design

Patients completed three surveys:

- Survey One: Expectation Assessment
  This five-point Likert scale survey was completed prior to treatment, after the patient’s treatment plan had been finalized. This survey collected information about the patient’s expectations of the proposed dental treatment relating to function, aesthetics, comfort and tissue preservation.
- Survey Two: SCL­90­R (non-patient norm females and males) [10]
  This survey was completed at the same time as Survey One and contained the 90 item SCL-90-R questionnaire that measured the current point in time psychological status as detailed in Table 1. The SCL-90-R normally takes 10–12 min to complete.
- Survey Three: Outcome Assessment
  This five-point Likert scale survey was completed at least 2 weeks after the completion of treatment to align with the usual recall and timetable used in the ADH, and collected information about the patient’s satisfaction with treatment provided relating to function, aesthetics, comfort and tissue preservation.

**Table 1:** Psychological parameters investigated in the SCL-90-R questionnaire [10]

Parameter	Description
Somatization (SOM)	Reflects distress arising from perceptions of bodily dysfunction
Obsessive compulsive (O-C)	Focuses on thoughts, impulses, and actions that are experienced as unremitting and irresistible and of an ego-alien or unwanted nature
Interpersonal sensitivity (I-S)	Feelings of inadequacy and inferiority, particularly in comparison with other people
Depression (DEP)	Reflects a representative range of the manifestations of clinical depression. Symptoms of dysphoric mood and affect are represented as are signs of withdrawal of life interest, lack of motivation, and loss of vital energy
Hostility (HOS)	Reflects thoughts, feelings, or actions that are characteristic of a negative affected state of anger.
Anxiety (ANX)	General signs of anxiety such as nervousness, tension and trembling as well as panic attacks and feeling of terror, apprehension and dread
Phobic Anxiety (PHOB)	Persistent fear response to a specific person, place, object or situation that is irrational and disproportionate to the stimulus and leads to avoidance or escape behavior
Paranoid Ideation (PAR)	Represents paranoid behavior fundamentally as disordered mode of thinking
Psychoticism (PSY)	Represents the construct as a continuous dimensionand reflects reflect a graduated continuum from mild social alienation to first-rank symptoms of psychosis
Global Severity Index (GSI)	The average rating given to the 90 items of psychological symptoms and represents the overall psychological distress level
Positive Symptom Distress Index (PSDI)	The average rating given to those symptoms that are self-reported (ie. not rated ‘0’) and represents the intensity of symptoms
Positive Symptom Total (PST)	Measures the number of self-reported symptoms that were rated higher than ‘0’

Patients completed the survey anonymously and responses to the three surveys were linked by a unique, de-identified survey number. Each patient was provided with a full explanation of the dimensions as well as the methods of scoring each questionnaire.

### Data collection

The SCL-90-R raw dimension scores were standardized by age and sex to provide a T-score for each dimension. Using the hand scoring worksheet, raw scores from each subscale were converted to standardized T-scores to enable comparisons of the psychological traits. Each subscale was compared for each outcome variable. Data were recorded and analysed using SASv9.4 software (SAS Institute Inc., Cary, NC, USA).

### Statistical analysis

Psychological variables were summarised as means with standard deviations and medians with interquartile ranges (Q1-Q3). The satisfaction and expectation measures were converted into binary variables due to small cell counts on all measures and dichotomised to represent strongly agree (5 = “1”) versus the remaining categories (1–4 = “0”). This was clinically reasoned to delineate patients who were completely satisfied (strongly agree = 1) and needed no further intervention from patients who had at least some concerns (all other scores = 0) who would require at least some type of remedial management.

Change scores were not calculated as different wording was used to assess patient expectation and satisfaction scores. Logistic regression models were used to assess the impact of psychological variables on patient satisfaction. Adjustment was made for baseline expectations in all models as this was an accepted alternative to modelling change scores in pre- and post-assessment studies. A “*p*” value < 0.05 was considered statistically significant.

## Results

All patients (*n* = 30) completed each of the three surveys. There were equal numbers of male and female patients, with four patients aged 20–40, 13 aged 41–60, and 13 aged 61–80 years.

Composite restorations (*n* = 16) were the most frequently completed treatment followed by crowns (*n* = 9), composite restoration with a crown (*n* = 3), composite restoration with bleaching (*n* = 1), and composite restoration with a veneer (*n* = 1). No patients received treatment to replace a missing tooth. The treatment was provided by undergraduate students (*n* = 25), postgraduate students (*n* = 4) and a prosthodontist (*n* = 1).

### Chewing satisfaction (Table 2)

Chewing satisfaction was not significantly associated with any of the baseline psychological measures.

**Table 2:** Association of chewing satisfaction with baseline psychological measures

Variable	Odds Ratio	Confidence Interval (95%)	***P*** Value
**Expect**	1.19	0.641–2.21	0.583
**SOM**	0.98	0.92–1.05	0.563
**O-C**	0.99	0.93–1.04	0.632
**I-S**	1.03	0.95–1.11	0.501
**DEP**	0.96	0.90–1.03	0.252
**ANX**	1.01	0.95–1.07	0.795
**HOS**	0.99	0.93–1.06	0.859
**PHOB**	1.01	0.93–1.11	0.752
**PAR**	1.02	0.95–1.09	0.646
**PSY**	0.99	0.94–1.06	0.837
**GSI**	0.99	0.94–1.06	0.828
**PSDI**	0.97	0.89–1.07	0.552
**PST**	1.01	0.95–1.07	0.818

### Aesthetic satisfaction (Table 3)

Aesthetic satisfaction was significantly associated with psychoticism (*p* = 0.032) and positive symptom distress index (*p* = 0.027). In both cases, increasing scores were associated with poorer outcomes as indicated by a reduction in the odds of stating “strongly agree”. Following adjustment for baseline aesthetic expectations, each unit increase in psychoticism score was associated with an 8% reduction in the odds of strongly agreeing with the statement “I like the appearance of the teeth that have been treated” (Odds Ratio (OR) = 0.92; 95% CI: 0.86–0.99) and a reduction of 14% for positive symptom distress (OR = 0.86; 95% CI: 0.75–0.98). The odds ratio represented each unit increase in the psychological scale scores being associated with a decreasing likelihood of strongly agreeing with the statement after adjusting for baseline expectation.

**Table 3:** Association of aesthetic satisfaction with baseline psychological measures

Variable	Odds Ratio	Confidence Interval (95%)	***P*** Value
**Expect**	0.54	0.19–1.54	0.246
**SOM**	0.91	0.83–1.00	0.059
**O-C**	0.94	0.88–1.01	0.084
**I-S**	0.95	0.87–1.04	0.265
**DEP**	0.95	0.88–1.02	0.155
**ANX**	0.96	0.89–1.03	0.220
**HOS**	0.97	0.90–1.04	0.341
**PHOB**	0.95	0.87–1.04	0.276
**PAR**	0.96	0.89–1.03	0.294
**PSY**	0.92	0.86–0.99	0.032
**GSI**	0.95	0.88–1.02	0.152
**PSDI**	0.86	0.75–0.98	0.027
**PST**	0.96	0.89–1.03	0.260

### Comfort satisfaction (Table 4)

Comfort satisfaction was significantly associated with the obsessive compulsive score (*p* = 0.034), depression (*p* = 0.037) and anxiety (*p* = 0.034). In all cases, a one unit increase in the scale score was associated with an 8% reduction in the odds of strongly agreeing with the statement “My teeth feel comfortable and natural” (obsessive compulsive OR = 0.92; 95% CI: 0.86–0.99; depression OR = 0.92; 95% CI: 0.85–1.00; anxiety OR = 0.034; 95% CI: 0.84–0.99).

**Table 4:** Association of comfort satisfaction with baseline psychological measures

Variable	Odds Ratio	Confidence Interval (95%)	***P*** Value
**Expect**	0.89	0.47–1.65	0.704
**SOM**	0.97	0.90–1.04	0.390
**O-C**	0.92	0.86–0.99	0.034
**I-S**	0.92	0.84–1.01	0.094
**DEP**	0.92	0.85–1.00	0.037
**ANX**	0.92	0.84–0.99	0.034
**HOS**	0.97	0.91–1.04	0.353
**PHOB**	0.95	0.87–1.05	0.324
**PAR**	0.97	0.90–1.04	0.355
**PSY**	0.95	0.88–1.01	0.098
**GSI**	0.94	0.87–1.01	0.073
**PSDI**	0.93	0.84–1.03	0.188
**PST**	0.94	0.87–1.01	0.099

### Tissue preservation satisfaction (Table 5)

Following adjustment for baseline expectation, tissue preservation satisfaction was associated with somatization (*p* = 0.024), obsessive compulsive (*p* = 0.032), interpersonal sensitivity (*p* = 0.026), depression (*p* = 0.014) and global severity index (*p* = 0.033). In all cases, a one unit increase on the scale score was associated with a reduction in the odds of strongly agreeing with the statement “overall, I am satisfied with my treatment”.

**Table 5:** Association of tissue preservation satisfaction with baseline psychological measures

Variable	Odds Ratio	Confidence Interval (95%)	***P*** Value
**Expect**	0.73	0.29–1.85	0.512
**SOM**	0.85	0.74–0.98	0.024
**O-C**	0.91	0.84–0.99	0.032
**I-S**	0.85	0.74–0.98	0.026
**DEP**	0.89	0.80–0.98	0.014
**ANX**	0.93	0.87–1.01	0.077
**HOS**	0.98	0.92–1.05	0.544
**PHOB**	0.98	0.89–1.07	0.597
**PAR**	0.95	0.89–1.03	0.219
**PSY**	0.95	0.89–1.02	0.164
**GSI**	0.89	0.81–0.99	0.033
**PSDI**	0.91	0.81–1.02	0.111
**PST**	0.92	0.85–1.00	0.058

## Discussion

SCL-90-R is a useful psychological testing instrument containing nine primary symptoms including somatization, obsessive compulsive symptoms, depression, anxiety, psychoticism, paranoid ideation, interpersonal sensitivity, phobic anxiety and hostility. The components are more diverse in comparison with other instruments, such as NEO-FFI.

Our study found that significant associations were found between SCL-90-R subscale scores for somatization, obsessive compulsive symptoms, depression, psychoticism, anxiety and interpersonal sensitivity with the treatment outcome, with increasing dissatisfaction associated with increasing SCL-90-R subscale scores. The somatization finding corroborates previously reported positive correlations between somatization and orofacial pain and denture incompatibility [2, 3].

A positive relationship was found between dissatisfaction with aesthetic dental treatment and obsessive compulsive symptoms that could reasonably be attributed to a tendency toward perfectionism especially in the aesthetic zone. The psychoticism subscale was also associated with the treatment outcome. Psychoticism symptoms have varying effects on the level of function but often include prolonged symptoms of progressive social withdrawal, poor self-care, auditory hallucinations, delusions and impaired concentration [6]. In our study, these patients had a high tendency for dissatisfaction despite the quality of the aesthetic treatment provided probably because they were predisposed to experience negative effects. As expected, chewing satisfaction was not significantly associated with any of the baseline psychological measures which suggested function was not one of patient’s main objective in aesthetic restorative treatment.

Although the treatment expectations for the aesthetic, comfort, function and tissue preservation components were high, patients generally reported high levels of satisfaction on all measured treatment outcomes. This could be attributed to the treatment being carried out in a university setting in a well-controlled teaching environment where each clinical step is checked by a qualified and experienced clinical tutor. Alternatively, there is no real way to know the extent to which subjects were “eager to please” for a range of possible reasons. The findings are limited to adult patients receiving treatment in the public sector and avoids potential problems of overlap with care provided through private practice where cost may influence outcomes.

The findings of the present study provide useful information to assist clinicians in identifying and understanding patients who may have a high risk of dissatisfaction with aesthetic dental treatment. More specifically, in the present study environment, the psychological testing instrument has a potential role in assigning patients to the most appropriate clinic for treatment as well as use a primary health care screening for depression in primary health care settings [11].

It has been demonstrated that patient expectations of treatment may be higher than dentist expectations, for example in relation to the ‘whiteness’ of teeth [12]. Whiter teeth have been positively correlated with high levels of social competence, intellectual ability, psychological adaptation and sociability and is frequently reported as the most important factor in determining satisfaction with self-appearance [13]. Discrepancies between patient expectations and the result of treatment is a key reason for dissatisfaction of aesthetic restorative treatment and underscores the importance of matching patient treatment expectations to treatment outcomes before treatment commences [14].

Previous research established certain personality traits are associated with satisfaction with some dental treatments [15]. A relationship was found between complete and partial removable prosthodontic rehabilitations and daily living satisfaction, dental satisfaction and patient personality profiles [16]. Patient total satisfaction and satisfaction with appearance, pain tolerance, oral comfort, and eating after treatment were higher than that before treatment [16]. However, the personality assessment revealed neuroticism scores displayed a negative linear relationship to satisfaction with appearance.

The diagnosis of a psychological disorder with the SCL-90-R alone was not possible and was not the objective of this study. Nevertheless, the SCL-90-R is a reliable psychometric test and incorporated gender-keyed norms that evaluated the current psychological symptom expression of the included patients [9]. Despite the relatively widespread use of the SCL-90-R testing instrument, the use of the instrument in this study was not validated in the study population. Also, there was potential for patient satisfaction to be influenced by the type of clinician, there was no clinician calibration, and there was no method to standardise the actual treatment time for different treatments conducted for individual patients.

The sample size of this study was limited. The study was designed to determine the potential for profiling of this type and the focus on aesthetic dental treatment limits the application of the findings. Studies with larger sample sizes and involving changes in psychological status at different stages throughout a variety of different clinical treatments and including patients from the public and private sector will reveal the full potential of this approach. The study is also limited by the small sample size which in part was due to patients’ reluctance to participate in our study. Reluctance to participate in such studies may be due to the stigma associated with psychologically-related research, which has been reported in previous studies [17]. Despite the low number of participants in our study, the study did provide useful early information that would direct further investigation in this area.

Further analysis may reveal additional psychological trait dimensions that could contribute to negative treatment satisfaction for specific treatments. For example, most patients with dental anxiety problems have been shown to have multiple other psychological problems such as somatization, psychoticism and general psychoneuroticism [18]. Future investigations could be expanded to explore the nature, extent and clinical implications of the relationship between treatment expectations, psychological traits and the prediction of treatment outcomes with greater sample sizes.

## Conclusions

Within the limitations of this preliminary study, the SCL-90-R shows initial promise in assisting clinicians to identify and understand patients who have a high risk of dissatisfaction with aesthetic dental treatment. The psychological dimension of patients presenting for dental treatment should be assessed whenever dental needs are assessed.

## Data Availability

The datasets used and/or analysed during the current study are available from the corresponding author on reasonable request and in line with ethical responsibilities.

## Abbreviations

ADH: Adelaide Dental Hospital
NEO-FFI: NEO Five-Factor Inventory
SCL-90-R: Symptom Checklist-90-Revised
